# On the Multimodal Path to Language: The Relationship Between Rhythmic Movements and Deictic Gestures at the End of the First Year

**DOI:** 10.3389/fpsyg.2021.616812

**Published:** 2021-02-10

**Authors:** Eva Murillo, Ignacio Montero, Marta Casla

**Affiliations:** ^1^Departamento de Psicología Básica, Facultad of Psicología, Universidad Autónoma de Madrid, Madrid, Spain; ^2^Departamento de Psicología Social y Metodología, Facultad de Psicología, Universidad Autónoma de Madrid, Madrid, Spain; ^3^Departamento de Psicología Evolutiva y de la Educación, Facultad de Psicología, Universidad Autónoma de Madrid, Madrid, Spain

**Keywords:** gestures acquisition, multimodalily, deictic gestures, rhythmic movement, language development

## Abstract

The aim of this study is to analyze the relationship between rhythmic movements and deictic gestures at the end of the first year of life, and to focus on their unimodal or multimodal character. We hypothesize that multimodal rhythmic movement performed with an object in the hand can facilitate the transition to the first deictic gestures. Twenty-three children were observed at 9 and 12 months of age in a naturalistic play situation with their mother or father. Results showed that rhythmic movements with objects in the hand are a frequent behavior in children's repertoires. Rhythmic behaviors tend to decrease from 9 to 12 months, specifically when they are unimodal. Multimodal rhythmic behavior production at 9 months is positively related with proximal deictic gestures 3 months later. Multimodal rhythmic movements are not directly related to distal deictic gestures, but are indirectly related via proximal deictic gestures. These results highlight the relevance of multimodal behaviors in the transition to the use of early gestures, and can be considered as a transitional phenomenon between the instrumental action and early communicative gestures.

## Introduction

The link between oral and motor components evolves continuously from the very early hand and mouth connections of the newborn to the adult speech and gesture synchrony (Iverson, 2010), bringing out the multimodal nature of language (Perniss, 2018). This study focuses on how this link is present in the rhythmic movements performed by infants at the end of the first year, and how multimodal rhythmic movements can be a transitional path from instrumental action to early gestures with a clear communicative goal. We hypothesize that the multimodal rhythmic movement performed with an object in the hand can pave the way to the use of the first deictic gestures. Our proposal is based on four sources of evidence.

The first one comes from the Dynamic Systems Theory, and specifically, from Iverson and Thelen's (1999) model of gesture and speech coordination development. Iverson and Thelen (1999) propose four phases in the development of the coupling system between gestures and speech. The first phase, from birth to the first 6 months of life, is denominated “*initial linkages*.” In this phase, we can observe the Babkin reflex in newborns, as well as the hand-to-mouth behavior and the tendency of infants to bring objects to their mouths. In the second phase, “*emerging control*” (approximately from 6 to 8 months), there is an increase in rhythmical movements of the arms and the hands. In this phase, canonical babbling emerges. Both phenomena appear to be related in this period, since manual rhythmic movements tend to occur with canonical babbling and vice versa (Ejiri and Masataka, 2001; Iverson and Fagan, 2004; Iverson et al., 2007). To explain this coordination, Iverson and Thelen (1999) propose that the two motor systems (the hands and the jaw) mutually influence one another. Ultimately, they settle on a “compromise” frequency at which they entrain to produce a coordinated behavior.

The next phase, called “*flexible coupling*,” lasts from 9 to 14 months. In this period, rhythmic movements of the hands and arms tend to decrease and canonical babbling gives way to word-like productions. Rhythmic repetition is replaced by more controlled communicative resources and communicative gestures emerge. However, little is known about how rhythmic movements are progressively replaced by movements that have a clear communicative intention, such as gestures.

The last phase described by Iverson and Thelen is denominated “*synchronous coupling*.” In this phase, we can observe the emergence of synchronous speech and gesture. According to Iverson and Thelen, it is during this period when speech and gesture begin to be synchronous, and this coupling remains stable throughout the child's early development and in adult language. However, some studies have shown that the synchrony between vocal and gestural components is present before the first words (Esteve-Gibert and Prieto, 2014; Murillo et al., 2018) and is related to subsequent lexical achievements (Murillo et al., 2018).

According to Iverson and Thelen's model, the use of multimodal communicative gestures would not be established until the last phase. The focus of the present study is on the transition between the “flexible coupling” and the “synchronous coupling” phases, or in other words, on the relationship between rhythmic movements and subsequent communicative gesture development.

The second source of evidence comes from the Pragmatics of the Object perspective (Rodríguez and Moro, 1998). From this point of view, the adult is a mediator between the child and the world, and the object is considered as a communicative tool. Although traditionally the beginning of triadic interactions is placed at the end of the first year, adults include objects in the interactions with their infants from the first months of life (Rodríguez et al., 2015). These interactions are not triadic in the sense of the children intentionally communicating about an object, but they could be considered as more “basic” triadic interactions, given that the adult includes the child and the world in a communicative act (Moreno-Núñez et al., 2017). From the first months of an infant's life, adults often make a rhythmic use of the objects in their interactions (Moreno-Núñez et al., 2015). The rhythmic structure of the early social interactions helps the infants to give structure to the interaction itself and, in a broader sense, to their experience in the world (Moreno-Núñez et al., 2015). As infants develop, adults progressively let them take the lead in these rhythmic interchanges (Moreno-Núñez et al., 2017; Aureli et al., 2018). Therefore, during the first months of life, infants are familiar with how these rhythmic interchanges work, as well as with the social impact of these actions on the adult.

The third source of evidence has to do with the dichotomy between action and gesture. Gestures have a communicative goal, that is, they are produced with the intention of conveying a meaning to another person. However, it is not always evident when infants start to perform gestures with a clear communicative intention (Donnellan et al., 2020). As Andrén (2014) claims, instead of defining “the lower limit of gesture” as a dichotomy, there are dimensions, such as communicative explicitness or representational complexity, that can help us to make decisions about what is in fact considered a gesture. According to these dimensions, multimodal rhythmic movements would have a low level of representational complexity. Regarding communicative explicitness, rhythmic movements are actions framed in focused interactions with ambiguous communicative status. Thus, according to Andrén (2014), they would have an intermediate level of communicative explicitness. This view opens up the possibility of considering rhythmic movements as a transitional phenomenon, more than a simple instrumental action on the objects. This is relevant given the fact that from a traditional communicative perspective, they do not reach the criteria for being considered as a communicative gesture. Some of the abilities needed to establish a shared reference (McCune and Zlatev, 2015), such as the ability to maintain conscious attention to some entity or event, and the ability to draw the attention of an audience to this entity or event voluntarily, are already present when children perform multimodal rhythmic movements in the context of social interaction. The multimodal rhythmic movements would be in a middle point between instrumental and communicative actions. The rhythmic interactions in which the child and the adult both give their attention to the same object can set the basis for subsequent joint attention abilities. As claimed by Clark (1996), the most direct form of joint attentional frame or “common ground” is perceptual co-presence, that is, when both adult and infant are perceptually attending to something and they are mutually aware that they are. Multimodal rhythmic interactions can help build this “common ground” or joint attentional frame that is essential for identifying the intended reference of deictic gestures (Tomasello et al., 2007).

Finally, the last source of evidence comes from studies emphasizing the consideration of language as an inherently multimodal phenomenon (Perniss, 2018). Regarding language development, there is growing evidence highlighting how different elements that precede verbal development such as gestures and prosody are synchronized and predict later linguistic abilities (see Hübscher and Prieto, 2019, for a review). The multimodal features of language facilitate different linguistic achievements throughout the language development process.

Early multimodal communicative behaviors have a predictive value on subsequent linguistic milestones. The relationship between vocal and gestural coordination and different lexical, syntactic, and morphological skills through the early stages of language development is well-known. When the vocal element is not a word yet, gesture and vocalization combinations at 12 months of age predict lexical development 3 months later (Murillo and Belinchón, 2012; Wu and Gros-Louis, 2014) and even at 18 months (Igualada et al., 2015). Children initially produce constructions that coordinate gesture and speech with similar structures to those that they will produce later when combining words (Özçalişkan and Goldin-Meadow, 2005, 2009). Moreover, the age at which children start producing pointing + noun combinations predicts the onset age for determiner + noun constructions (Cartmill et al., 2014). The onset of two-word combinations can be predicted depending on the onset of gesture and word combinations, in which each element conveys a different meaning (supplementary coordination) (Butcher and Goldin-Meadow, 2000; Goldin-Meadow and Butcher, 2003; Iverson and Goldin-Meadow, 2005; Capobianco et al., 2017). The production of gesture and speech combinations at 22 months of age also predicts sentence complexity when children are 42 months old (Rowe and Goldin-Meadow, 2009). It seems, therefore, that the coordination of vocal and gestural elements is present throughout the language development process.

As language skills develop, multimodal patterns evolve, providing structures on which children build later abilities. The relationship between distal deictic gestures, especially pointing, and later linguistic abilities has been widely documented (see Colonnesi et al., 2010, for a meta-analysis). Similarly, proximal deictic gestures, in which the object remains in contact with the hand, precede and are related to distal deictic gestures (Cameron-Faulkner et al., 2015). Recent findings suggest that the declarative motive of infant pointing can be found at 10 months in holdout gestures, that is, in a proximal deictic gesture (Boundy et al., 2019), and by 11 months of age, showing gesture coordinated with gaze is a strong predictor of language development by 15 months (Donnellan et al., 2020).

Putting all these pieces together, we find that before the emergence of the first communicative gestures (that is, actions produced with the intention of conveying a meaning to another person), caregivers include the objects in the interactions and make a rhythmic use of them, and by doing this, they give structure to the interaction itself. This structure helps enable children to progressively take the lead in these rhythmical exchanges and promote the perception of contingencies in the social interaction. During the transition from rhythmic movements to more controlled communicative forms, children have to learn how to draw adults' attention toward a proximal object (e.g., showing objects) and then they learn how to direct adults' attention toward something out of reach by means of distal deictic gestures. Multimodal rhythmic behaviors can be an intermediate step between the instrumental action and the communicative gestures and can set the basis for joint attention interchanges. Our study focuses on the transition from the rhythmic movements produced by the end of the third trimester of life to the emergence of the first communicative gestures. Our research question is whether the rhythmic movements produced with an object in the hand are related to the first deictic gestures: proximal deictic gestures, which are aimed at drawing adults' attention to the object held in the hand. We wanted to explore whether a higher rate of production of multimodal rhythmic movements with objects at 9 months of age is related with a higher rate of proximal deictic gestures at 12 months.

Our hypothesis is that multimodal rhythmic movements can have an intermediate communicative status between the instrumental actions with the object and the deictic gestures. We hypothesize that multimodal rhythmic movements produced with an object in the hand will be related to early proximal deictic gestures. This hypothesis does not involve a causal relationship between the two phenomena, but proposes that the social experience derived from rhythmic multimodal movements (that is, the sociopragmatic skills developed by multimodal means in a multimodal context, as described by Hübscher and Prieto, 2019) can be useful for learning to intentionally manage others' attention in triadic interactions. According to this, the rate of multimodal rhythmic movements with objects produced at 9 months will be related to the increasing in proximal multimodal deictic gestures production from 9 to 12 months.

All in all, we expect to find that by the end of the first year, infants will frequently produce rhythmic movements with objects in their hand in social interactions, although these will tend to give way to more explicit and controlled communicative gestures. These rhythmic movements will often be accompanied by vocalizations, constituting multimodal patterns. The first deictic gestures such as give and show (or holdouts) are produced with the object in the hand (proximal deictic gestures), thus we hypothesize that multimodal rhythmic movements with objects produced at 9 months of age will be specifically related to proximal deictic gestures 3 months later. Multimodal rhythmic gestures could be a previous step to the transition to the first communicative gestures.

## Method

### Participants

Twenty-three children participated in the study (13 girls, 10 boys) with their mother or father. Families were contacted through several day care facilities. The participants all came from monolingual Spanish-speaking homes and they were all full term, had uncomplicated pregnancies, and normal deliveries. Before every observation session, we administered the Spanish version of the Battelle Developmental Screening test (De la Cruz and González, 1996) to all the participants, so as to ensure that their acquisition of motor milestones was within the normal range, and that they all followed a typical development. The Mean equivalent age from the Battelle Screening test was 8 months and 4 days (SD = 1 month, 2 days) for the 9-month observation session and 11 months and 21 days (SD = 2 months, 16 days) for the 12-months observation session.

The parents agreed to participate voluntarily and provided informed consent. The University Research Ethics Committee approved all the procedures in the study.

The children were observed interacting with a primary caregiver in a spontaneous situation playing with objects at two different times. First, when they were 9 months old (*M* = 9 months, 4 days; *min* = 8 months, 9 days; *max* = 9 months, 23 days; *SD* = 8.7 days) and 3 months later, when they were 12 months of age (*M* = 12 months, 8 days; *min* = 11 months, 9 days; *max* = 13 months, 2 days; *SD* = 9.6 days). The observation sessions were conducted in their homes or in an isolated room in their daycare center and were video recorded for further analysis. A camcorder was fixed to a tripod at ~2 m from the dyad. Parents were asked to play with their children as they usually do. We provided them with a set of toys including blocks, balls, cups, plates and spoons, a picture book, and a doll, but they could use any other toy present in the room if they wanted to. The mean session duration was 13 min. and 9 s (*min* = 7'33”; *max* = 18'30”; *SD* = 3'03”) when children were 9 months old, and 13 min and 21 s (*min* = 4'58”; *max* = 18'15”; *SD* = 3'03”) when children were 12 months old.

### Coding and Analysis

We coded infants' rhythmic behaviors, vocalizations, and deictic gestures using the ELAN software (Lausberg and Sloetjes, 2009), that allows a precision of 20 ms for coding. We coded the rhythmic behaviors produced with the arms or hands, adapting Thelen's (1981) criteria for defining the bouts of rhythmic movements (that is, the frequency of production) regardless of their duration. In our view, it is not how long is the child maintaining a rhythmic movement that could have a communicative impact, but the inclusion of elements that make it more intentionally communicative at the adult's eyes. We considered a motor behavior as rhythmic when it was repeated at least two consecutive times in the same form and at a regular pace. The rhythmic behavior ended when the infant's arms or hands returned to the resting position or when they performed a different behavior.

We coded a rhythmic behavior as “with object” when the child produced this movement with an object in the hand or banged an object with the hand or with another object. We considered a rhythmic movement as “without object” when the movement was produced with no object in the hands, i.e., the child shakes her hand(s) or arm(s) or banged a surface with the hand(s).

Regarding gestures, proximal and distal deictic gestures were coded as follows:

- Point: index finger visibly extended with some extension of the arm.
- Reach: the arm is extended with hand open and fingers straight.
- Show: the child holds up the object, but it remains in the child's possession.
- Give: infant hands object to adult and object changes hands.
- Other: any gesture observed not included in the previous categories or not clearly observable.

As previously mentioned, proximal deictic gestures are those deictics in which the object referred to remains in contact with the hand, that is, “show” and “give” gestures. Distal deictic gestures direct attention without holding the object in the hand. In our classification this includes “point” and “reach.”

These categories were adapted from previous studies, and more detail on coding categories can be found in Murillo and Belinchón (2012) and Murillo et al. (2018).

Regarding vocalizations, we coded all the vocal sounds produced by infants except vegetative sounds (e.g., hiccups). We considered it two different vocalizations when there was a second of silence or a conversational turn between them. We classified the children's vocalizations according to the following categories, adapted from Murillo and Belinchón (2012) and Murillo et al. (2018):

- *Babbling*: the utterance is not similar to any word of the language. It has no sound-meaning regularity and no formal relationship with the referent alluded.
- *Word*: the utterance is clearly identifiable as a word and has a referential sense. We included in this category the onomatopoeic sounds and the protowords, that is, utterances with a stable phonetic structure and a clear relationship with the referent but do not constitute a word in the adult language.
- *Other:* any vocal sound that is unclear or that cannot be included in the previous categories.

Two independent observers coded 5% of the recordings, with an inter-observer agreement of *k* = 0.92 for gestures (*n* = 40); *k* = 0.78 for vocalizations (*n* = 47); and *k* = 0.78 (*n* = 26) for rhythmic movements. After coding the observational categories, we considered as multimodal those behaviors with some temporal overlap between the motor component (gesture or rhythmic movement) and the vocal component or when the motor component was produced a second before or after the vocalization. We took a one-second window to consider as multimodal those behaviors in which vocalization or rhythmic movement started immediately before or after the other (Donnellan et al., 2020). When there was no temporal overlap between components, the communicative behaviors were considered as unimodal.

## Results

In order to analyze the production of rhythmic movements and how they evolve with age in relation to multimodality and the use of objects, we conducted a repeated measures ANOVA. We took the rate per minute of rhythmic movement production as the dependent variable. Multimodality (multimodal vs. unimodal), the use of object (with object vs. without it), and the age (9 vs. 12 months) were the factors. Table 1 shows the rate per minute of rhythmic movement production with and without object at 9 and 12 months of age.

**Table 1:** Mean (and standard deviation) of the rate per minute of rhythmic movements performed with and without object at 9 and 12 months.

			**Age**	
			**9 months**	**12 months**
			**Mean (SD)**	**Mean (SD)**
Rhythmic movements	Unimodal	With object	0.65 (0.46)	0.38 (0.44)
		Without object	0.17 (0.17)	0.13 (0.17)
	Multimodal	With object	0.17 (0.20)	0.16 (0.24)
		Without object	0.11 (0.26)	0.07 (0.10)

We found a significant three-way interaction effect [*F*(1, 22) = 4.45; *p* = 0.046; η^2^ = 0.16; *1–*β = 0.52], so we proceeded to analyze lower level interactions as recommended by Heiman (1995). Regarding multimodality, for unimodal rhythmic behaviors, we found a main effect of the object use [*F*(1, 22) = 24.005; *p* < 0.001; η^2^ = 0.52; *1–*β = 0.997], with more unimodal rhythmic behaviors performed with an object in the hand than without it. We also found a main effect of age, [*F*(1, 22) = 5.74; *p* = 0.026; η^2^ = 0.207; *1–*β = 0.629] with more unimodal rhythmic movements at 9 months than at 12 months.

Considering age, at 9 months, we found an interaction effect between multimodality and object use [*F*(1, 22) = 21.20; *p* < 0.001 η^2^ = 0.49, *1–*β = 0.99]. When rhythmic movements were produced with an object, there were more unimodal rhythmic movements than multimodal ones. At this age, unimodal rhythmic movements were more frequently produced with object than without it. There was no differences in object use when rhythmic movements were multimodal.

At 12 months, there was no interaction effect between object use and multimodality [*F*(1, 22) = 2.44; *p* = 0.133]. We found more unimodal than multimodal rhythmic movements [*F*(1, 22) = 6.66; *p* = 0.017; η^2^ = 0.23; *1–*β = 0.69] and more rhythmic movements produced with object than without it [*F*(1,22) = 9.40; *p* = 0.006; η^2^ = 0.30; *1–*β = 0.83].

When we considered rhythmic movements produced with an object, we found an interaction effect between age and multimodality [*F*(1,22) = 5.70*; p* = 0.026; η^2^ = 0.20; *1–*β= 0.62]. At 9 months, there was a higher rate of unimodal rhythmic movement produced with object than multimodal ones. At 12 months, the pattern was similar, but the differences between unimodal and multimodal rhythmic movement production were lower than those found at 9 months.

We also analyzed the evolution of proximal and distal deictic gestures in the period studied. Table 2 shows the mean rate of deictic gesture production at 9 and 12 months.

**Table 2:** Mean rate per minute, standard deviation, and range of multimodal and unimodal proximal and distal deictic gestures produced at 9 and 12 months of age.

			**Age**	
			**9 months**	**12 months**
			**Mean (SD) min–max**	**Mean (SD) min–max**
Deictic gestures	Unimodal	Proximal	0.10 (0.17) 0–0.62	0.20 (0.35) 0–1.35
		Distal	0.06 (0.08) 0–0.34	0.18 (0.32) 0–1.57
	Multimodal	Proximal	0.01 (0.03) 0–0.14	0.20 (0.26) 0–1.08
		Distal	0.009 (0.02) 0–0.09	0.12 (0.19) 0–0.71

We conducted a repeated measures ANOVA taking as a dependent variable the rate per minute of deictic gesture production. The modality (multimodal vs. unimodal), the type of deictic (proximal vs. distal), and the age (9 vs. 12 months) were the factors. Results showed a main effect of age [*F*(1, 22) = 9.76; *p* = 0.005; η^2^ = 0.30; *1–*β = 0.84], with more deictics produced at 12 months than at 9 months (0.18 vs. 0.046). There was no main effect of the type of deictic [*F*(1, 22) = 1.13; *p* = 0.29] neither of the modality [*F*(1, 22) = 3.38; *p* = 0.079]. We found no interaction effect between factors, suggesting that all deictics tend to increase with age in the period studied regardless of whether they are unimodal or multimodal.

**Figure 1:**
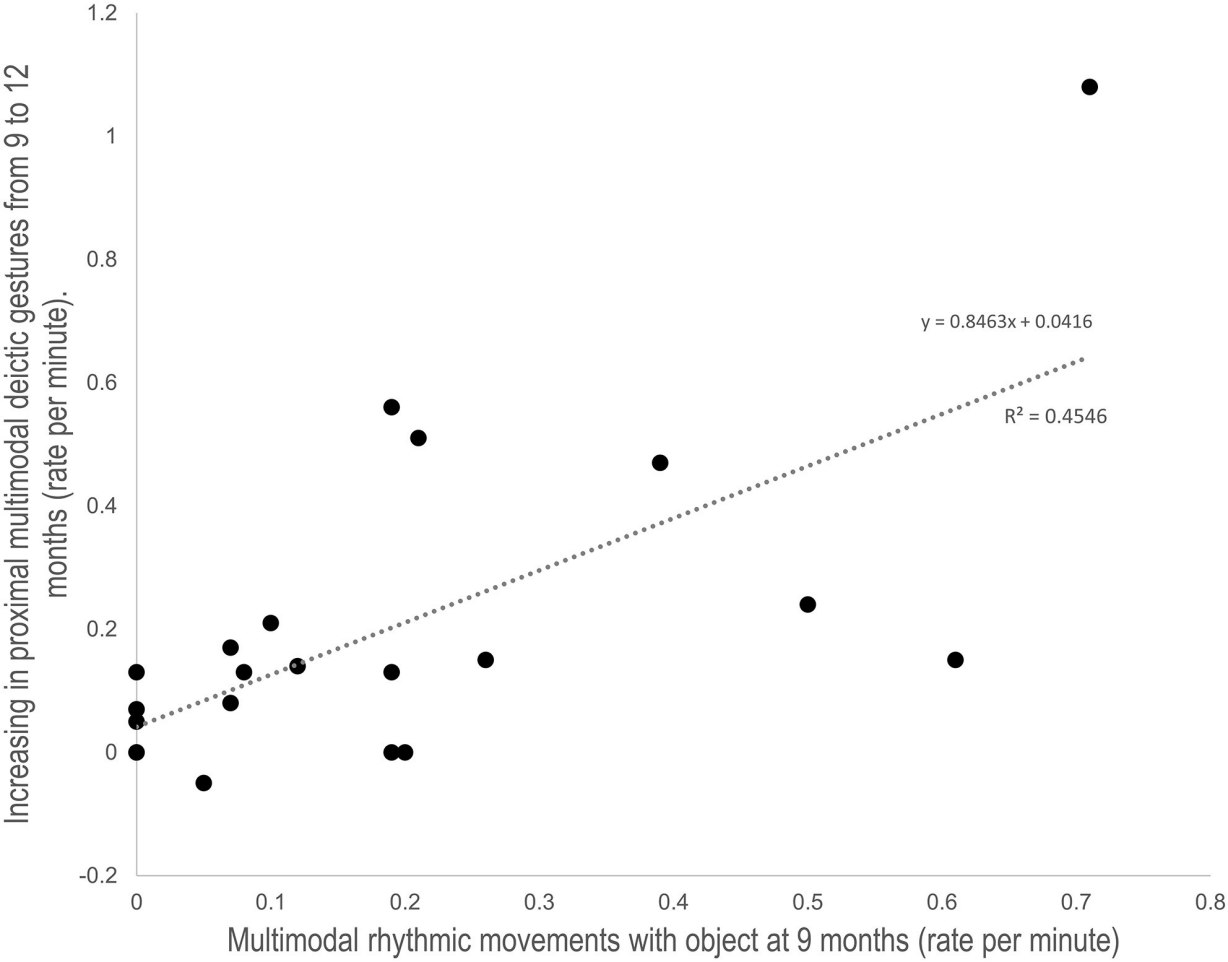
Scatterplot of the relation between the rate per minute of multimodal rhythmic movement production at 9 months and the increasing in the rate per minute of multimodal proximal deictic gestures between 9 and 12 months.

Finally, we explored the relationship between rhythmic movements and deictic gestures. Indeed, we wanted to test whether the frequency of multimodal rhythmic gestures produced with objects at 9 months were specifically related to the increase in proximal multimodal deictic gestures from 9 to 12 months. With this aim, we conducted a multiple stepwise regression analysis, taking the increase between 9 and 12 months in the rate per minute of proximal multimodal deictic gestures as dependent variable. We took the rate per minute of multimodal rhythmic movements with object, unimodal rhythmic movement with object, proximal multimodal gestures, proximal unimodal gestures, distal multimodal gestures, and distal unimodal gestures at 9 months as predictor variables. Results showed that the best predictor was the rate per minute of multimodal rhythmic gestures with object at 9 months (β = 0.67, *p* < 0.001), that explained 45% of the variance of the increase in the rate per minute of proximal multimodal deictic gestures from 9 to 12 months [F(1, 22) = 17.42; *p* < 0.001]. The inclusion of any of the other predictive variables did not significantly improve this prediction. Figure 1 shows the relationship between multimodal rhythmic movements at 9 months and the increase in proximal multimodal gestures in the period studied.

The frequency of multimodal rhythmic movements with an object at 9 months could be related to the increase in deictics production regardless of whether they were proximal or distal. To explore the relationship between rhythmic movements and deictic gestures produced at 9 months and the increase in multimodal distal deictic gestures from 9 to 12 months, we conducted the same analysis as described before, but taking the increase from 9 to 12 months in distal multimodal deictics production as dependent variable. In this case, rhythmic movements were not related to the increase in distal multimodal deictics but the best predictor was the rate per minute of proximal multimodal deictic gestures produced at 9 months (*R*^2^ = 0.17). The predictive value of the model increases with the inclusion of the frequency of distal unimodal deictic gestures production at 9 months (β = –0.38, *p* < 0.05). In this case, both variables predict 32% of the variance of the dependent variable [F(2, 20) = 4.87; *p* < 0.05; Δ*R*^2^ = 0.15]. It is worth noting that the correlation between unimodal distal gestures at 9 months and the increase in distal multimodal deictic gestures at 12 months is negative. According to this, the children that experience a higher increase in multimodal distal gestures are those that at 9 months of age produced more often proximal multimodal gestures and less distal unimodal ones.

These results highlight the specific relationship between multimodal rhythmic movements with objects and proximal multimodal deictic gestures, as well as the relevant role of multimodality in the relationship between rhythmic movements and deictic gestures development.

## Discussion

The aim of the present study was to analyze the evolution of rhythmic movement production taking into account their unimodal or multimodal character as well as the use of objects by infants during these rhythmical interchanges. Our results showed that the production of rhythmic movements in a social context is a frequent behavior in an infant's repertoire at the end of the first year.

We found more unimodal than multimodal rhythmic movements. However, unimodal rhythmic behaviors tend to decrease with age, especially when produced with an object in the hand, whereas there was no decrease for multimodal rhythmic behaviors. According to Iverson and Thelen (1999), the dynamic coupling of two effector systems (in this case limbs and oral structures) requires relatively high levels of activation in order for mutual entrainment to occur. This could explain the initial prevalence of unimodal rhythmic behaviors over the multimodal ones. As the child gains control over both systems, the activation threshold drops, increasing the probability of producing multimodal rhythmic behaviors. As Iverson and Thelen propose, rhythmic movements decrease in this period, but this decrease is mainly due to the reduction in the rate of unimodal rhythmic movements: whereas unimodal rhythmic behaviors decrease significantly from 9 to 12 months, the production of multimodal rhythmic behaviors remains stable.

On the other hand, deictic gesture production increases in the period studied as expected. When analyzing the relationship between rhythmic movements and deictic gestures, we found that multimodality takes on special importance. Following Iverson and Thelen's view, language is a motor-vocal system, and multimodality is a key feature of this system. The gesture-vocal coupling is present in language development even before the verbal elements emerge (Esteve-Gibert and Prieto, 2014; Murillo et al., 2018). The coordinated use of gestures and vocal elements are predictive of subsequent linguistic achievements. Murillo and Belinchón (2012) showed that the coordinated use of pointing, vocalizations, and social gaze at 12 months of age was a better predictor of lexical development 3 months later than any of the elements taken separately. Wu and Gros-Louis (2014) also found a similar relationship between vocalizations and pointing around 12 months and lexical development at 15 months. Similar results were reported by Igualada et al. (2015) showing that children increased their production of speech and gesture combinations to achieve a communicative goal, and that the use of gestures and speech combinations by 12 months of age were predictive of subsequent linguistic achievements at 18 months. Children point with vocalizations at 12 months, and when they have a declarative purpose, they attune the prosodic contour by 15 months of age (Aureli et al., 2017). Our results support this idea of language as a motor-vocal system, showing that the coordinated use of rhythmic actions and vocal elements precedes and is related to the first deictic gestures. The multimodal rhythmic movements can serve as an opportunity for the child to learn how to bring another person's attention toward a reference, which is precisely the goal of deictic gestures. Adults are more willing to react to children's behaviors when vocalizations are included (Balog and Brentari, 2008; Fasolo and D'Odorico, 2012; Ger et al., 2018). The coordinated use of rhythmic movements and vocalizations in a social context can facilitate the pragmatic development needed to perform the first communicative gestures. As Hübscher and Prieto (2019) claim, children's development of pragmatic skills is essentially multimodal and the multimodal characteristics of language facilitate children's socio-pragmatic development. Multimodal rhythmic movements performed with an object in the hand allow the children to experience with the attention toward the object held in hand and the adult's reaction to these movements as part of the same social interaction. Multimodal rhythmic movements can help to establish a shared reference or at least an initial “common ground” in Clark's (1996) sense.

Although rhythmic movements are not directly related to distal deictic gestures in our sample, they are indirectly related by means of multimodal proximal deictics. Although at first glance the negative relationship of unimodal distal gestures at 9 months and the increase in multimodal distal gestures may be surprising, it must be taken into account that both pointing and reaching gestures are included within the distal gestures category. Reaching gestures precede pointing in development, so it may be that a significant part of the unimodal distal gestures produced at 9 months were reaching gestures. On the other hand, the increase in distal gestures between 9 and 12 months could reflect the increase in the use of pointing that has been observed in this period in previous studies (Murillo and Belinchón, 2012; Murillo et al., 2018).

Donnellan et al. (2020) have shown that 11-month-old children produce proximal deictic gestures intentionally much more frequently than distal ones. They also found that showing gestures produced intentionally are related to later language development. Our results support Donnellan et al.'s (2020) idea that the configuration of showing (and by extension of proximal deictic gestures) allows infants to attend to both an object of interest and to the attention of the adult to that object, which plausibly scaffolds the transition to later triadic communication.

Our results can contribute toward filling a gap in the understanding of how multimodal behaviors which include motor and vocal elements develop to support the language learning process. However, our study has some limitations that must be taken into account. First of all, the sample size is limited. Second, we did not analyze adults' reactions to rhythmic movements in order to investigate a possible differential reaction which would depend on the multimodality and the use of objects. Since adults react more often to children's behaviors when they include vocalizations (Balog and Brentari, 2008; Fasolo and D'Odorico, 2012; Ger et al., 2018), further research is needed to clarify if this is the case regarding rhythmic movements. Third, extending the follow-up study to 15 months would have allowed us to analyze the changes in multimodality as well as the consolidation of distal deictic use and the maintenance of the links between rhythmic movements, proximal deictics, and distal deictics. Finally, the context of observation, with many objects available to the infant, could have prompted the child to produce the rhythmic movements with an object in the hand. However, we consider that this is likely to occur in infants' daily routines and the behaviors produced would be similar to those found in naturalistic settings. Despite these limitations, the results of our study shed light on the relationships between multimodal rhythmic movements and the use of communicative gestures and we feel this is an area to be studied in an even greater depth. Finally, the changes in the relationships between motor and vocal elements in interactive situations can provide clues with which to assess communicative development at the early stages of the language learning process.

## Data Availability Statement

The raw data supporting the conclusions of this article will be made available by the authors, without undue reservation.

## Ethics Statement

This study was reviewed and approved by the Ethics Committee of the Universidad Autónoma de Madrid (ref.: CEI-101-1896). Written informed consent to participate in this study was provided by the participants' legal guardian/next of kin.

## Author Contributions

EM designed the study. EM and MC collected the data. IM analyzed the data. All authors discussed the results and contributed to the final version of the manuscript.

## Conflict of Interest

The authors declare that the research was conducted in the absence of any commercial or financial relationships that could be construed as a potential conflict of interest.
